# Optic Nerve Sheath Diameter Ultrasound: A Non-Invasive Approach to Evaluate Increased Intracranial Pressure in Critically Ill Pediatric Patients

**DOI:** 10.3390/diagnostics12030767

**Published:** 2022-03-21

**Authors:** Giulia Cannata, Stefano Pezzato, Susanna Esposito, Andrea Moscatelli

**Affiliations:** 1 Pediatric Clinic, University Hospital, Department of Medicine and Surgery, University of Parma, 43126 Parma, Italy; cannata.giulia@gmail.com; 2 Intensive Care Unit, IRCCS Istituto Giannina Gaslini, 16147 Genoa, Italy; stepezza@gmail.com (S.P.); andreamoscatelli@gaslini.org (A.M.)

**Keywords:** intracranial hypertension, intracranial pressure, optic nerve sheath diameter, pediatric emergency, point-of-care ultrasound

## Abstract

Early diagnosis of increased intracranial pressure (ICP) is crucial for prompt diagnosis and treatment of intracranial hypertension in critically ill pediatric patients, preventing secondary brain damage and mortality. Although the placement of an external ventricular drain coupled to an external fluid-filled transducer remains the gold standard for continuous ICP monitoring, other non-invasive approaches are constantly being improved and can provide reliable estimates. The use of point-of-care ultrasound (POCUS) for the assessment of ICP has recently become widespread in pediatric emergency and critical care settings, representing a valuable extension of the physical examination. The aim of this manuscript is to review and discuss the basic principles of ultra-sound measurement of the optic nerve sheath diameter (ONSD) and summarize current evidence on its diagnostic value in pediatric patients with ICP. There is increasing evidence that POCUS measurement of the ONSD correlates with ICP, thus appearing as a useful extension of the physical examination in pediatrics, especially in emergency medicine and critical care settings for the initial non-invasive assessment of patients with suspected raised ICP. Its role could be of value even to assess the response to therapy and in the follow-up of patients with diagnosed intracranial hypertension if invasive ICP monitoring is not available. Further studies on more homogeneous and extensive study populations should be performed to establish ONSD reference ranges in the different pediatric ages and to define cut-off values in predicting elevated ICP compared to invasive ICP measurement.

## 1. Introduction

Timely detection and treatment of elevated intracranial pressure (ICP) are essential for preventing secondary brain damage and related morbidity and mortality [1,2,3,4,5]. Increased intracranial pressure can emerge as a result of both neurological and non-neurological diseases. Traumatic brain injury (TBI) and its complications, including increased intracranial pressure, are the leading causes of mortality and morbidity in children [6,7,8,9]. Other causes of increased ICP include intracranial infections, stroke, intracranial hemorrhage, hydrocephalus, ventricular shunt malfunction, brain tumors, arachnoid cysts, craniosynostosis syndromes, impaired central nervous system venous outflow, idiopathic intracranial hypertension, or hepatic encephalopathy [6,7,8,9]. Secondary brain injury occurs within minutes to hours of the primary injury due to a pathophysiologic cascade of events reducing perfusion, oxygen and metabolite delivery, and clearance of metabolic waste and toxins [10,11,12,13].

Monitoring ICP is crucial in managing critically ill neurological patients. The gold standard for accurate ICP monitoring is the invasive positioning of ventricular or intraparenchymal devices. Among these, the external ventricular drain (EVD) coupled to an external fluid-filled transducer remains the best choice both for its measurement accuracy and for allowing therapeutic CSF drainage at the same time [14,15,16,17,18,19,20,21] Several non-invasive ICP monitoring techniques have been attempted repeatedly [22,23,24,25]. The use of point-of-care ultrasound (POCUS) for diagnostic assessment has recently become widespread in pediatric emergency and critical care services, representing a valuable extension of the physical examination [26,27,28,29]. The intraorbital portion of the optic nerve, ontogenetically part of the central nervous system, extends from the ocular bulb to the optic canal and is surrounded by cerebrospinal fluid and optic nerve sheath (ONS), a membrane continuous with the dura mater of the brain. The perioptic subarachnoid space is a prolongation of the intracranial subarachnoid space, specifically, the chiasmal cistern; as the ONS is distensible, acute variations of cerebrospinal fluid pressure determine changes occurring within minutes in optic nerve sheath diameter (ONSD) [30,31,32,33,34,35,36].

Optic nerve sheath diameter ultrasound has been shown to correlate with increased ICP, thus appearing as a promising non-invasive and radiation-free bedside tool to assess elevated ICP [34,37,38,39,40,41]. Optic nerve sonography has been applied in a variety of pediatric diseases at risk for intracranial hypertension, including: traumatic and nontraumatic brain injury, intracranial hemorrhage, diabetic ketoacidosis, metabolic disorders (hepatic failure), ventriculoperitoneal shunts, hydrocephalus, suspected intracranial lesions, hypoxic injury, meningoceles, spina bifida and craniosynostosis [34,42,43,44,45,46,47,48,49].

The aim of this narrative manuscript is to review and discuss the basic principles of ultrasound measurement of the ONSD and summarize current evidence on its diagnostic value in pediatric patients with ICP.

## 2. Pathophysiology of Raised Intracranial Pressure (ICP)

**Figure 1:**
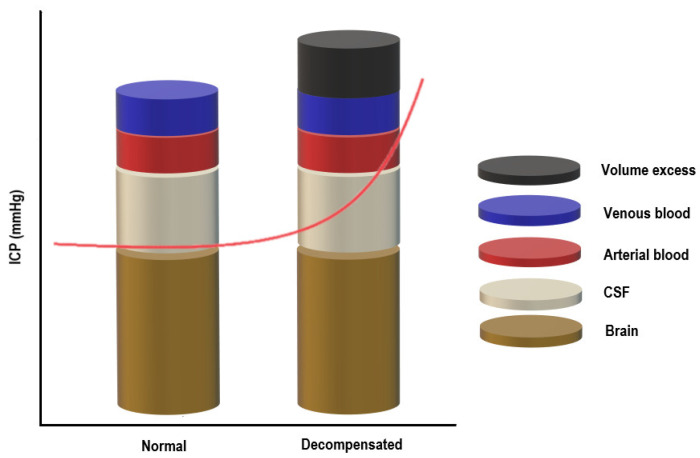
The Monro–Kellie doctrine.

The skull can be imagined as a rigid box containing the following components: brain tissue, cerebrospinal fluid, and blood (arterial and venous). The Monro–Kellie model of ICP states that for ICP to remain constant, the sum of the volumes of the components mentioned above should remain constant [50,51]. Since brain tissue is assumed to be incompressible due to its high-water content, there must be a balance between the inflow and outflow of the intracranial fluids to keep ICP stable [52]. CSF secretion must be equal to the absorption rate, and at the same time, the arterial cerebral blood flow has to equal the effluent venous drainage (Figure 1) [50,51].

Under normal conditions, the intracranial volume is constant, and maintaining a steady ICP depends on the volume of the intracranial compartments (brain + CSF + blood): an increase in one component will cause a compensatory decrease in one or both of the others [53,54]. Raised ICP can result from any pathological condition increasing the volume of any of the three components or from the addition of a fourth component (e.g., intracranial hemorrhage, cerebral edema, or mass), overwhelming the compensatory mechanisms. Once the reserve is exhausted, the intracranial compliance will decrease, and slight elevations in the intracranial volume will lead to dramatic changes in ICP [53,54]. CO_2_, O_2_ and blood vessels size influence ICP in the critically ill patient [53,54].

Cerebral blood flow (CBF) is driven by cerebral perfusion pressure (CPP), which is defined as mean arterial pressure (MAP) minus intracranial pressure (CPP = MAP−ICP). Cerebrovascular autoregulation (CA) is tightly linked to CPP. It refers to the capacity of the cerebral circulation to alter the vascular arteriolar resistance to maintain a constant CBF as mean arterial pressure (MAP, and thus CPP) varies. In healthy adults, CA is normally operational across a wide range of MAPs, from 50 to 150 mm Hg. Beyond the limits of autoregulation, CBF becomes pressure passive. Few data are available in the pediatric population.

On the other end of the equation, ICP elevations can compromise the CPP leading to secondary ischemic brain injury. In the face of high ICP, brain ischemia can be partially counteracted by increasing the MAP through manipulation of the cardiac output and arterial pressure. Increased ICP can further compromise the brain parenchyma through herniation syndromes [55,56,57,58,59,60,61]. ICP fluctuates under physiologic conditions, including body posture (orthostatism vs. clinostatism), cardiorespiratory variations, electroencephalography (EEG) activity, and changes of the intrathoracic (ITP) and intra-abdominal pressure (IAP; if central venous pressure exceeds ICP) [62,63,64,65,66,67]. ICP is referenced at the level of the foramen of Monro. The normal suggested reference values for ICP vary with age (Table 1) [68,69,70,71,72].

It is currently accepted that physiologic mean ICP boundaries in healthy adult subjects resting in the horizontal position are 7–15 and −10 mm Hg but not exceeding −15 mm Hg in the vertical position. Normal mean ICP values are reported to be within the range of 3–7 mm Hg in young children and 1.5–6 mm Hg in term infants [68,69,70,71,72].

The definition of intracranial hypertension depends on each specific clinical condition. Acute intracranial hypertension (AIH) in adults has been classically defined as sustained ICP above 20 mm Hg for greater than five minutes. An ICP treatment threshold of 20 mm Hg is used in children since there is sufficient evidence in pediatric literature suggesting an association between ICP values greater than 20 mm Hg and poor outcome [6,15,68,73,74,75,76,77,78,79].

## 3. Optic Nerve and Its Measurement

### 3.1. Anatomy and Physiology of Optic Nerve

The intraorbital portion of the optic nerve, ontogenetically part of the central nervous system, extends from the ocular bulb to the optic canal and is surrounded by cerebrospinal fluid and optic nerve sheath (ONS), a membrane made up of leptomeninges in continuity with the dura mater of the brain.

The optic nerve is approximately 40 mm long and 4 mm wide, including the sheath, with an average diameter of 0.4 mm. The subarachnoid space features a structure of arachnoidal trabeculae, septa, and stout pillars. Under normal conditions, it holds approximately 0.1–0.2 mL of cerebrospinal fluid [30,80,81,82,83,84,85].

**Figure 2:**
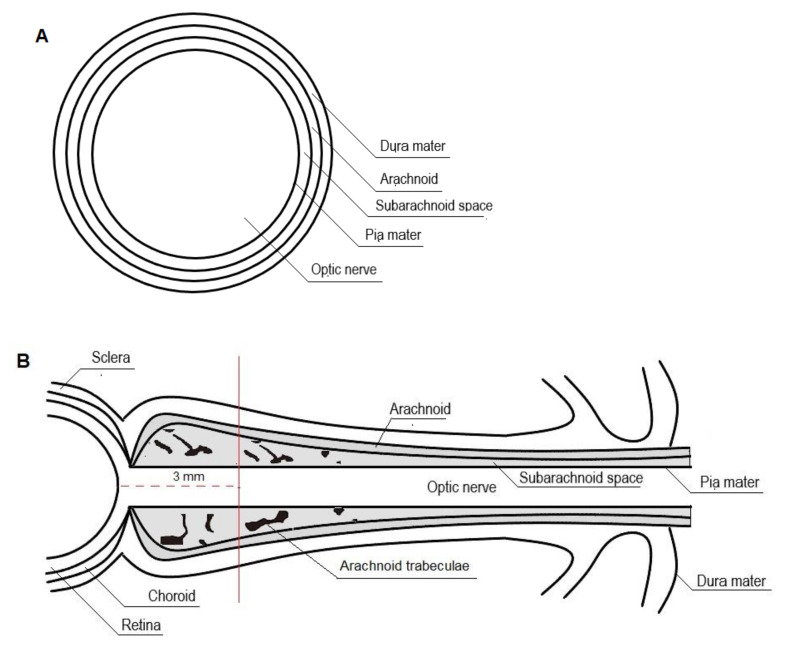
Cross-section (**A**) and longitudinal section (**B**) of the optic nerve.

The perioptic subarachnoid space is a prolongation of the intracranial subarachnoid space, specifically, the chiasmal cistern [83,84,86,87]. It has been hypothesized that the perioptic CSF slowly percolates toward the bulbar portion of the nerve and that reversal flow occurs with eye movements squeezing the retrobulbar ONS (Figure 2) [30,84,86].

As the ONS is distensible, optic nerve sheath diameter (ONSD) changes rapidly with changing cerebrospinal fluid pressure. The ONSD is constant as long as the ICP remains within normal ranges. When ICP rises, maximum ONSD fluctuations occur in the anterior subarachnoid compartment, 3 mm behind the globe, rather than in the posterior perineural one. It has been suggested that this non-uniform enlargement may be the result of the asymmetrical distribution of the arachnoidal trabeculae, with lower density in the retrobulbar ONS. Moreover, the anterior compartment of the ONS is the thinnest of the entire segment and, therefore, the most distensible [30,31,33,88,89,90,91,92].

### 3.2. Ultrasonographic Technique for Optic Nerve Sheath Diameter (ONSD) Measurement

Optic nerve sonography is performed with a high-frequency linear transducer (>7.5 MHz), the patient lying supine, with the head in a neutral position and both eyes closed.

The probe is gently placed in an axial plane on the temporal side of the closed upper eyelid using a thick layer of sterile coupling ultrasound gel (lateral transbulbar approach). B-mode is selected. A transverse sonographic section allows for visualization of the globe and the structures of the retrobulbar area, including the optic nerve in its longitudinal course [32,39,47,89,93,94,95,96,97,98,99,100].

On images, the optic nerve complex is shown as a homogenous hypoechoic band extending posteriorly from the bulb′s base in the context of the echogenic retrobulbar fat. More specifically, the OND appears as a hypoechogenic structure surrounded by the more hyperechogenic ONSD, but still hypoechogenic compared to the retrobulbar fat. Color Doppler may be used to facilitate optic nerve identification through visualization of the central retinal artery and vein running inside [94,101].

**Figure 3:**
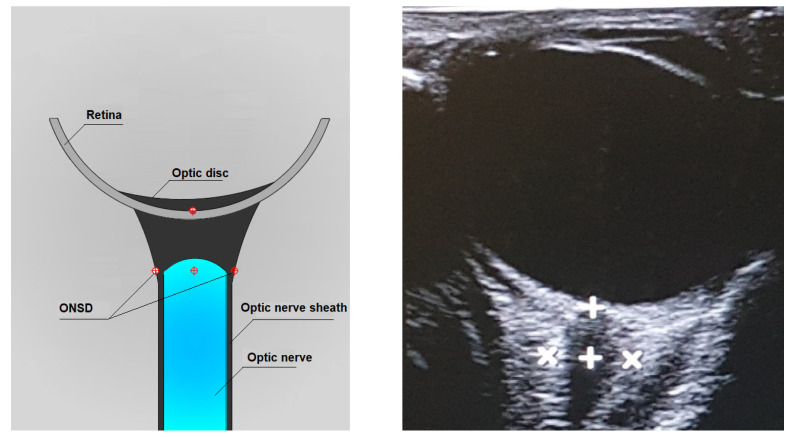
Axial lateral transbulbar approach ONSD measurement.

By convention, the ONSD measurement is performed 3 mm posterior to the papilla base by manual cursor placement on the outer contours of the optic nerve sheath. The zoom feature can be helpful for the correct display of the cursors (Figure 3) [30,32,33].

## 4. Optic Nerve Sheath Diameter (ONSD) Measurements in Children

Knowledge of normal pediatric ONSD reference ranges is essential for detecting raised ICP in clinical practice.

Optic nerve sheath diameter reference ranges in children and neonates were first established in 1999 by Ballantyne et al. A total of 102 children aged from 0 to 15 years were included in the study; none suffered from neurological or ophthalmological disease. Optic nerve sheath diameter data were grouped by age: the range of normal values in children under 1 year was 2.1–4.0 mm, and the range for children over 1 year of age was 2–3 mm. The cut-off value for abnormal enlargement was above 4 mm in infants under 1 year of age and 4.5 mm in older children [102].

Rehman Siddiqui et al., identified an ONSD ultrasonographic threshold predictive of elevated ICP in various age groups [103]. Forty-eight children aged from one month to 16 years with the following inclusion criteria were enrolled in the study: traumatic brain injury defined as moderate (Glasgow coma scale 9–13) or severe (Glasgow coma scale <9), clinical signs and symptoms suggestive of raised ICP, progressive neurological deterioration and active malignancy history with new onset of neurological symptoms. Patients diagnosed with orbital trauma with orbital fractures, orbital tumors, or intraocular space-occupying lesions were excluded from the study. Cut-off ultrasonographic value for abnormal ONSD enlargement predictive of raised elevated ICP was above 4 mm in infants, 4.71 mm in children aged 1–10 years, and 5.43 mm in older children [103].

A total of 13 patients aged between 12 and 18 years as candidates for an elective lumbar puncture with the suspicion of idiopathic intracranial hypertension (IIH) were enrolled in the prospective study conducted by Irazuzta et al. [104]. Patients underwent ONSD ultrasound examination while awake immediately before sedation. A complete concordance was observed between the cut-off value for raised ICP (cerebrospinal fluid opening pressure above 20 cmH_2_O) and ONSD measured by ultrasonography (*p* < 0.01). An ONSD of >4.5 mm correlated with an increased ICP (sensitivity 100%, *p* < 0.01). Patients without elevated ICP had an ONSD <4.5 mm (specificity 100%) [104].

Aslan et al., evaluated the correlation between lumbar puncture opening pressure and ultrasonographic ONSD measures in seven patients diagnosed with pseudotumor cerebri syndrome (PTCS) [105]. This condition is characterized by raised ICP with no neuroradiological abnormalities. The control group included a total of 15 healthy children.

In the PTCS group, ultrasonographic ONSD) values of both eyes were statistically significantly higher than in the control group (*p* < 0.001). They also showed a significant correlation between the lumbar puncture opening pressure and ONSD baseline measures for both the right and the left eye (*r* = 0.882, *p* = 0.009 and *r* = 0.649, *p* = 0.004, respectively) [105].

Padayachy et al., analyzed the diagnostic accuracy of ONSD cut-off values compared to invasive ICP measurement at thresholds of 20, 15, 10, and 5 mm Hg in different age groups and taking into account the patency of the anterior fontanelle [106]. Data from 174 patients <14 years of age under general anesthesia were analyzed.

In children ≤1 year old, the ONSD measurement with the best diagnostic accuracy for detecting ICP ≥20 mm Hg was 5.16 mm (SE 80% (44.4–97.5), SP 76.1% (61.2–87.4), PPV 42.1% (20.3–66.5), NPV 94.6% (81.8–99.3), 95% CI). In children >1 year old, the ONSD measurement with the best diagnostic accuracy for detecting ICP ≥20 mm Hg was 5.75 mm (SE 85.9% (75–93.4), SP 70.4% (56.4–82), PPV 77.5% (66–86.5), NPV 80.9% (66.7–90.9), 95% CI) [106].

Likewise, Kerscher et al., compared ONSD cut-off values with invasively measured ICP values [107]. A total of 72 patients were enrolled in the study; 40% were investigated under general anesthesia, 39% were awake, 14% were sedated for lumbar or shunt reservoir puncture, and 7% were somnolent or comatose in the intensive care unit. Diagnostic accuracy of ONSD cut-off values have been compared to ICP measurement at thresholds of 5, 10, 15, 20, 25, and 30 mm Hg in different age groups and considering the patency of the anterior fontanelle. In children ≤1 year old, the ONSD measurement for detecting ICP ≥20 mm Hg was 4.99 mm (SE 50%, SP 58.8%, PPV 22.2%, NPV 83.3%); in children >1 year old was 5.75 mm (SE 91.7%, SP 66.7%, PPV 45.8%, NPV 96.3%). The authors also showed a significant correlation between ONSD values and intracranial pressure for children >1 year (*r* = 0.63, *p* < 0.01). The correlation was poor for patients ≤1 year (*r* = 0.21; open anterior fontanelle: *r* = 0.057, closed anterior fontanelle: *r* = 0.4) [107].

Robba et al., compared different non-invasive ultrasound-based methods of ICP evaluation with simultaneous direct readings from invasive ICP monitoring devices (either intraparenchymal or intraventricular catheters) [108]. A total of 10 children aged <16 years with an indication for invasive ICP monitoring were enrolled in the study. Among the non-invasive methods studied, ONSD ultrasound presented the best accuracy to assess ICP: ONSD measurements correlated with invasive ICP values (*r* = 0.852, *p* < 0.0001). The ONSD measurement with the best diagnostic accuracy for detecting ICP ≥ 20 mm Hg was 4.75 mm (SE 0.956, SP 0.938, AUC 0.976—95% CI = 0.948–1.00); considering a threshold of 15 mm Hg, the ONSD measurement with the best diagnostic accuracy was 3.85 mm (SE 0.811, SP 0.939, AUC 0.94—95% CI = 0.892–0.989) [108].

Fontanel et al., investigated normal ultrasonographic ONSD values in children aged 0 to 18 years and created an optic nerve growth curve [109]. The authors also defined the accuracy of ONSD cut-off values according to age group for intracranial hypertension (IHT) diagnosis. Two hundred fifteen children underwent ONSD ultrasound examination. The enrolled patients were divided into three groups: 165 healthy children, 29 children diagnosed with IHT (all >4 years of age), and 21 children with optic disc drusen. Ultrasound examination was performed on awake patients. Exclusion criteria were optic nerve disorders potentially influencing the ONSD measurement (congenital coloboma, microphthalmos, inflammation of the optic nerve such as papillitis, and diseases associated with abnormal intraocular pressure such as glaucoma). The authors detected a statistically significant difference between ultrasonographic ONSD values of both eyes between healthy subjects and IHT subjects and between IHT subjects and subjects with optic disc drusen (*p* < 0.001). Optic nerve growth curve for healthy subjects showed a progressive increase in ONSD values up to approximately 10 years of age, and then ONSD values remained constant until the age of 18, with an upper limit of 4.5 mm. In children >4 years old and for the subgroup 4–10 years, the ONSD cut-off value was 4.1 mm (SE 100%, SP 83.9 and SE 100%, SP 89.3% respectively), and 4.4 mm for the subgroup 11–18 years of age (SE 100%, SP 98.8%) [109].

Table 2 summarizes ONSD reference ranges and cut-off values for predicting elevated ICP for the pediatric population reported by different studies [102,103,104,105,106,107,108,109,110,111,112].

## 5. ONSD Measurements in Neonates

Published data about ONSD ultrasound measurements in the neonatal population are limited and mainly based on studies with a small sample size.

The first study defining reference ranges in neonates and children was carried out in 1999 by Ballantyne et al. [102]: 102 children aged 0 to 15 years admitted for abdominal or hip ultrasound evaluation underwent ONSD ultrasound examination. None suffered from neurological or ophthalmological diseases.

Optic nerve sheath diameter ultrasound data were grouped by age: the range of normal values for ONSD in children under 1 year was 2.1–4.0 mm, and the range for children over 1 year of age was 2.4–4.3 mm. The cut-off value for abnormal enlargement was above 4 mm in infants under 1 year of age and 4.5 mm in older children [102].

Gravendeel et al., established reference values for ONSD ultrasound measurements in 120 (boys 65, girls 55) healthy full-term neonates (gestational age between 37–42 weeks), with a birth weight more than 2500 g and uncomplicated postnatal course [113]. Ultrasound examinations were performed within 1–4 days of delivery; follow-up ultrasound re-examination was carried out at 4 months and 8 months of age. The mean ONSD value with 95% reference intervals reported was 3.9 mm (3.1–4.7) in healthy term boys aged 1 to 2 days and 3.7 mm (2.7–4.7) in healthy term girls. ONSD measurements and reference intervals markedly increased between birth and 4 months [113].

Ardell et al., carried out a pilot study to document ranges of ONSD ultrasound measurements in preterm infants [114]. Twelve patients had weekly serial scans between 29 and 36 weeks of corrected gestational age; a total of 114 scans were performed on both eyes. No patients with suspected or confirmed raised ICP or intraocular pressure were included in the study. They showed a significant correlation between ONSD measurements and corrected gestational age. On the contrary, weight and head circumference did not correlate strongly with ONSD [114].

Yapicioglu et al., have recently published the largest database of ONSD normal values in preterm and term neonates [115]. Overall, 554 newborns without intracranial pathology were enrolled in the study. Detailed reference intervals are given for any different gestational ages.

Optic nerve sheath diameter measurements at 3 mm from the papilla were impossible in some of the preterm babies, since cursors fell beyond the longitudinal ultrasonographic section of the optic nerve; measurements at 2 and 2.5 mm were possible in some cases. Moreover, the authors showed a significant and positive correlation between ONSD measurements and gestational age and somatic parameters (weight, height, and head circumference) [115].

The optic nerve sheath diameter (ONSD) values for neonates reported by different studies are summarized in Table 3 [102,113,114,115].

## 6. Discussion and Future Directions

Increased ICP is a medical and often neurosurgical emergency. Real-time detection and dynamic evaluation of ICP in critically ill pediatric patients are crucial to warrant prompt diagnosis and management, thus preventing neurological morbidity and mortality [1,2,3,4,5].

Although the gold standard for ICP monitoring is invasive, with ventricular or intraparenchymal probes, the sonographic measurement of ONSD can provide valuable ICP estimates for the initial non-invasive assessment of patients with suspected raised ICP. Its role could be of value even to assess the response to raised ICP treatment and in the follow-up during the patient′s ICU stay and post-ICU care if more appropriate tools (invasive ICP monitoring) are not available or indicated [14,15,16,17,18,19,20,21,22,23,24,25].

In recent years, the use of point-of-care ultrasound (POCUS) for diagnostic assessment has become widespread in pediatric emergency and critical care medicine [26,27,28,29]. In comparison with conventional neuroimaging, such as magnetic resonance imaging (MRI) and computed tomography (CT), the advantages of POCUS of ONSD are low costs, short investigation times, repeatability, bedside availability, and radiation free. Contraindications to ONSD ultrasound examination are ocular trauma, diseases associated with abnormal intraocular pressure (e.g., glaucoma), optic nerve atrophy, and inflammatory lesions of the optic nerve affecting the ONSD [116].

However, in the current practice in pediatric neurocritical care, ONSD ultrasound appears to be underutilized because of several limitations affecting diagnostic accuracy in detecting raised ICP. Multiple studies reported significant variability in ONSD reference ranges and cut-off values predictive of elevated ICP in the different pediatric age groups. Most available data come from heterogeneous studies in terms of sample size, inclusion criteria (age, sex, somatic parameters, causes of increased ICP and comorbidities), reference standards (e.g., invasive ICP monitoring, CT, MRI, CSF OP at LP) and methodological approaches. Moreover, the ONSD ultrasonographic measurement technique should be standardized in the matter of probe lateral resolution and image acquisition [117,118,119]. By convention, the ONSD measurement is performed 3 mm posterior to the papilla base in adults. In pediatric patients, the maximal distensible part of the optic nerve sheath may be much more proximal than in adults, and further studies are needed. Furthermore, in some preterm ONSD, measurements 3 mm behind the papilla base are not always possible, as cursors could go beyond the longitudinal ultrasonographic section of the optic nerve [30,32,33,115,118].

Additional future research directions also include assessment of the learning curve and interobserver and intraobserver variability for POCUS of ONSD [120,121].

## 7. Conclusions

The optic nerve sheath diameter (ONSD) appears as a surrogate marker for the detection of raised ICP. POCUS of ONSD could represent a useful extension of the physical examination in pediatrics, especially in emergency medicine and critical care settings, for the initial non-invasive assessment of patients with suspected raised ICP. Repeat ONSD ultrasound measurements may be of value in an ICU setting to assess the response to raised ICP treatment and in the follow-up if invasive ICP monitoring is not available or indicated. Further studies on more homogeneous and extensive study populations should be performed to establish ONSD reference ranges in the different pediatric ages. The diagnostic accuracy of ONSD ultrasonographic cut-off values in predicting elevated ICP compared to invasive ICP measurement at different thresholds needs further investigation.

## Figures and Tables

**Table 1:** Suggested age-related intracranial pressure (ICP) reference values.

Population	ICP Reference Values
Adults	<10–15 mm Hg
Children	3–7 mm Hg
Term infants	1.5–6 mm Hg

Adapted from: Dunn LT. Raised intracranial pressure. J Neurol Neurosurg Psychiatry. 2002 September; 73 Suppl 1(Suppl 1): i23-7. doi:10.1136/jnnp.73.suppl_1.i23.

**Table 2:** ONSD values in the pediatric population: study characteristics.

Author	Included Children (n)	Normal Mean ONSD Value (mm)			Cut-Off Value for ONSD (mm)
Ballantyne et al. [102]	5 (0–2 months)	2.57 (SD 0.30)			>4 (<1 year)
	9 (2–3 months)	2.95 (SD 0.35)			>4.5 (1–15 years)
	5 (3–12 months)	3.21 (SD 0.22)			
	9 (1–2 years)	2.99 (SD 0.23)			
	17 (2–3 years)	3.03 (SD 0.20)			
	18 (3–4 years)	3.15 (SD 0.28)			
	16 (4–5 years)	3.23 (SD 0.38)			
	10 (5–10 years)	2.98 (SD 0.16)			
	13 (10–15 years)	3.26 (SD 0.35)			
Rehman Siddiqui et al. [103]	48	Patients with signs of raised ICP		Patients with no signs of raised ICP	
	8 (1 month–1 year)	4.64 (SD 0.48) *n* = 3		4.32 (SD 0.71) *n* = 5	>4 (SE 100% SP 60%)
	21 (1–10 years)	6.44 (SD 0.65) *n* = 10		5.03 (SD 0.82) *n* = 11	>4.71 (SE 100% SP 63.6%)
	19 (10–16 years)	6.28 (SD 0.62) *n* = 13		5.46 (SD 0.91) *n* = 6	>5.43 (SE 100% SP 66.7%)
Irazuzta et al. [104]	13 (12–18 years)	Patients with CSF OP greater than 20 cm _2_O		Patients with CSF OP less than 20 cm H_2_O	>4.5 mm (SE 100%)
		*n* = 3		*n* = 10	
		5.5 ± 1.2 (right eye)		3.9 ± 0.1 mm (right eye)	
		5.4 ± 1 (left eye)		3.7 ± 0.2 mm (left eye)	
Aslan et al. [105]	22 (7–17 years)	PTCS group		Control group	Not reported in the article
	(84–204 months)				
	PTCS group	*n* = 7		*n* = 15	
	(30–204 months)	6.7 (SD 0.5) (right eye)		5.3 (SD 0.2) (right eye)	
	Control group	6.7 (SD 0.6) (left eye)		5.2 (SD 0.3) (left eye)	
Padayachy et al. [106]	174	Not reported in the article			ICP threshold of 20 mm Hg
	Overall				5.5
	56 (≤1 year)				5.16 (SE 80%, SP 76.1%)
	118 (>1 year)				5.75 (SE 85.9%, SP 70.4%)
	62 (open AF)				5.16 (SE 85.7%, SP 75%)
	112 (closed AF)				5.80 (SE 85%, SP 73.1%)
Kerscher et al. [107]	72	Not reported in the article			ICP threshold of 20 mm Hg
	Overall				5.57 (SE 81.3%, SP 62.5)
	21 (≤1 year)				4.99 (SE 50%, SP 58.8%)
	51 (>1 year)				5.75 (SE 91.7, SP 66.7)
					ICP threshold of 15 mm Hg
	Overall				5.28 (SE 90.9%, SP 69.2%)
	21 (≤1 year)				4.99 (SE 71.4%, SP 71.4%)
	51 (>1 year)				5.57 (SE 80%, SP 69.2%)
Robba et al. [108]	10 (4–14 years)	3.70 (4.50–3.40) Median (IQR)			ICP threshold of 20 mm Hg
					4.75 (SE 0.956, SP 0.938)
					ICP threshold of 15 mm Hg
					3.85 (SE 0.811, SP 0.939)
Fontanel et al. [109]	215 (0–18 years)				
					≥4.1 (SE 100%, SP 83.9%)
	29 IHT				
	0 (<1 year)				
	0 (1–4 years)				
	29 (>4 years)	IHT	Healthy	ODD	
		*n* = 29	*n* = 165	*n* = 21	(>4 y)
	165 healthy				
	21 (<1 year)	4.9 (4.5–5.1) (>4 y)	4.0 (3.8–4.1) (>4 y)	4.0 (3.8–4.0) (>4 y)	≥4.1 (SE 100%, SP 89.3%)
	29 (1–4 years)				
	115 (>4 years)				
		Median (IQR)	Median (IQR)	Median (IQR)	≥4.4 (SE 100%, SP 98.8%) (11–18 y)
	21 optic disc drusen (ODD)				
	1 (<1 year)				
	1 (1–4 years)				
	19 (>4 years)				
Steinborn et al. [110]	81 (3–17.8 years)	Increased ICP		Normal ICP	>6 (SE 82%, SP 74%)
		*n* = 25		*n* = 56	
		6.85 (SD 0.81)		5.77 (SD 0.48)	
Malayeri et al. [42]	156	Case group		Control group	Not reported in the article
		*n* = 78		*n* = 78	
	34 (<4 years)	5.55 (SD 0.68) (<4 y)		3.00 (SD 0.67) (<4 y)	
	44 (>4 years)	5.68 (SD 0.71) (>4 y)		3.60 (SD 0.42) (>4 y)	
	Case group increased ICP				
	32 (<4 years)				
	46 (>4 years)				
	Control group normal ICP				
Aslan et al. [111]	57 (3–204 months)	Increased ICP		Normal ICP	5.8 (SE 66%, SP 100%)
		(suspected clinically or radiologically)			
		*n* = 38		*n* = 19	
		5.9 (SD 0.8)		5.2 (SD 0.3)	
Le et al. [112]	64 (0–18 years)	Increased ICP		Suspected ICP	>4 (<1 year)
	Suspected or confirmed increased ICP	(cranial imaging or direct measurement)			>4.5 (>1 year)
		*n* = 24		*n* = 40	(SE 83%, SP 38%)
		Not reported in the article		Not reported in the article	

SE, sensitivity; SP, specificity; AF, anterior fontanelle; OP, opening pressure on lumbar tap; CSF, cerebrospinal fluid; PTCS, pseudotumor cerebri syndrome.

**Table 3:** ONSD values in the neonatal population: study characteristics (SD, standard deviation).

Author	Included Neonates (n)	Normal Mean ONSD Value (mm)		Cut-Off Value for ONSD (mm)
Ballantyne et al. [102]	5 (0–2 months)	2.57 (SD 0.30)		>4 (<1 year)
	9 (2–3 months)	2.95 (SD 0.35)		>4.5 (1–15 years)
	5 (3–12 months)	3.21 (SD 0.22)		
	9 (1–2 years)	2.99 (SD 0.23)		
	17 (2–3 years)	3.03 (SD 0.20)		
	18 (3–4 years)	3.15 (SD 0.28)		
	16 (4–5 years)	3.23 (SD 0.38)		
	10 (5–10 years)	2.98 (SD 0.16)		
	13 (10–15 years)	3.26 (SD 0.35)		
Gravendeel J et al. [113]	120 (37–42 weeks of gestation)			Not reported in the article
	0–4 days	3.9 (3.1–4.7)		
	4 months	5.5 (4.5–6.5)		
	8 months	5.8 (5.0–6.6)		
		95% reference intervals Males		
	0–4 days	3.7 (2.7–4.7)		
	4 months	5.3 (4.3–6.3)		
	8 months	5.6 (4.6–6.6)		
		95% reference intervals Females		
Ardell S et al. [114]	12 preterm infants (29–36 weeks postconceptional age)	Right eye	Left eye	Not reported in the article
	29 weeks	2.1 (SD 0.1)	2.1 (SD 0.2)	
	30 weeks	2.2 (SD 0.1)	2.3 (SD 0.2)	
	31 weeks	2.4 (SD 0.1)	2.4 (SD 0.1)	
	32 weeks	2.7 (SD 0.3)	2.7 (SD 0.4)	
	33 weeks	2.7 (SD 0.2)	2.7 (SD 0.3)	
	34 weeks	2.9 (SD 0.2)	3.0 (SD 0.3)	
	35 weeks	3.2 (SD 0.4)	3.2 (SD 0.3)	
	36 weeks	3.1 (SD 0.3)	3.2 (SD 0.2)	
Yapicioglu et al. [115]	554			Not reported in the article
	22 (23 weeks 0 day–28 weeks 6 days)	2.6 (SD 0.3)—Distance 2 mm		
		2.7 (SD 0.3)—Distance 2.5 mm		
	64 (29 weeks 0 day–32 weeks 6 days)	3.0 (SD 0.2)—Distance 2 mm		
		3.1 (SD 0.2)—Distance 2.5 mm		
	167 (33 weeks 0 day–36 weeks 6 days)	3.3 (SD 0.2)—Distance 2 mm		
		3.5 (SD 0.2)—Distance 2.5 mm		
		3.6 (SD 0.2)—Distance 3 mm		
	301 (37 weeks 0 day–41 weeks 6 days)	4.0 (SD 0.2)—Distance 3 mm		

## Data Availability

Not applicable.
